# Annotations

**Published:** 1891-05-16

**Authors:** 


### Annotations.

**The Influenza Epidemic.**

The epidemic of influenza appears to be spreading rapidly. It is not of a very severe type. But *bonâ fide* influenza in any form requires to be managed with care and judgment because of its possible sequelæ. Perhaps the worst effect of the uncomplicated disease upon the system is the rapid and extreme exhaustion of the nervous energy. Probably most of the other unpleasant or dangerous consequences spring from this central cause. If this be so—and we are convinced it is—it follows that the chief object of treatment must be, in the first place, the careful conservation of the nervous energy by all possible means until the climax of the attack is reached, and after that the prompt restoration of the nervous energy to its normal level. Such being the aim of rational treatment, the patient must do all he can to "conserve his own energy" by resting absolutely, and resting in the recumbent posture, either in bed or in a well aired and properly warmed sitting-room. After rest, the next most important part of the the business is the feeding of the patient. The food should be sufficient, stimulating, nutritious, and light. It should be given, not in heavy meals three or four times a-day, but in small and often-repeated quantities. If the patient cannot eat, he should be fed with milk, whipped eggs, and all kinds of fluid or semi-fluid foods. Stimulants, alcoholic or otherwise, should be given frequently in small quantities. The bowels and other excretory organs should be kept in a state of normal activity. These, we are persuaded, are the right principles of treatment. Details as to drugs and food it is impossible to give. It is plain, however, that patients can do very much to help themselves; and the very best thing they can do is to prove themselves "patients" and not "impatients," by staying at home until the doctor gives them leave to go out. A good many people died of influenza last year, and a considerable number are dying now. Most of those who died owed their deaths to complications, which in many instances were brought about by their unwillingness to lay up for a sufficient length of time. It cannot be too often repeated that the only way of minimising the serious effects of influenza is to submit unreservedly to the imperious demands of the disease for a week or ten days. This submission at the outset often enables the patient to bid defiance to the later and much more dangerous sequelæ.

**The Medical Hat.**

Most of our readers may know that it has been proposed that all the coachmen of the Berlin doctors shall wear white hats, in order that the members of the general public may know a doctor's carriage immediately when they see it, and may thus be enabled to obtain skilled help at once in the sharpest emergency. The suggestion has commended itself pretty widely to that considerable class of persons who love to carry pins in the sleeves of their coats, and generally to make ample provision for all sorts of possible and impossible contingencies. But a far more improved and advanced suggestion comes from one of the *Lancet's* witty correspondents. That gentleman's letter is so exactly and entirely "on the business" that we give it in full: "I see," says he, "that the medical hat is again to the front. May I suggest that this elegant headgear would be rendered more effective at night by the simple addition of a luminous hat-band? The name, qualifications, and address of the wearer might be impressed in red thereon, thus proving useful to suffering humanity both by night and day." "C. Ch.," for that is the *nom de plume* which the writer makes use of, is evidently in full sympathy with the most advanced opinions of the period. The luminous hat-band, with the doctor's degrees and titles set forth at length thereupon, is admirable, though not quite original. Most of us have seen high-bred gentlemen of the "loafer" persuasion with their hats elegantly adorned and encircled by brown fly papers, to which innumerable unlucky flies adhered, giving proof positive of the efficacy of the article. The fly paper round the hat of the loafer is perhaps the parent of the luminous hatband. The only additional suggestion we can think of by way of making ideally complete that which the *Lancet's* correspondent brings so near to perfection, is, that the doctors should have a uniform professional crest and motto painted on their carriage panels, and that it should consist of a pill-box, with a surgery boy rampant, and the motto, "Not to be taken until the fee is paid." If, in addition, the horse or horses could be painted with alternate stripes of white zinc ointment and orange red rhubarb powder made into a suitable enamel, nothing would be wanting to the completeness and appropriateness of the equipment, or to the public facilities for securing a doctor without a moment's delay. The actual facts, however, seem to lie all the other way. Doctors, instead of being too scarce, are altogether too plentiful. It is quite a common thing for a family practitioner to find, when he is called in desperate haste to a strange patient, that half-a-dozen other practitioners have been sent for simultaneously, and that most of them are on the spot when he arrives. The white hat for doctors' coachmen is, in truth, quite unnecessary, and more than a little ridiculous. Let us hope that this new German advertising fever will not spread beyond the walls of Berlin.

**A General Practitioner of the Old School.**

There died at Greenwich last week Mr. Gay Shute, F.R.C.S., a general practitioner of the old school: "A fine old English gentleman, one of the olden times." Mr. Shute, who never wished to be called Dr. Shute, practised his profession at Greenwich for nearly half a century. He was born at Gosport on November 1st, 1812, and died on May 4th of the present year. There was not a more honourable or a more honoured man in Greenwich. He was a practitioner of first-rate capacity, and, needless to say, of almost unique experience. As a boy, Mr. Shute was educated at private schools, and well educated; as a young man he studied at University College Hospital and thence passed to the Chichester Hospital to be House Surgeon, remaining in the latter position for five years. Mr. Shute wanted nothing but ambition to have made him one of the most eminent surgeons of his time. He had all the eminence that Greenwich could give him in its best days; for he was for many years the chief figure among its medical practitioners by universal consent. But such were his courage, mental capacity, operative skill, nobility of presence, and rare goodness of heart, that if he had chosen the metropolis as the field of his active life he could not have failed to have taken one of the most commanding positions in the medical profession. There is nothing, however, to regret in the fact that Mr. Shute lived and died a general practitioner. He could not have served his generation in any position, however exalted, more honestly and capably than in that in which he actually lived and worked. He set an example of duty, honourable conduct and noble self respect to all the practitioners in Greenwich and for miles round it. He was well known and honoured by men of the highest medical eminence in London and the country. It will be an evil day for the medical profession when all the practitioners of the highest class shun general practice and seek to be consultants. A worthier, a more useful, and a more truly dignified career than that of Mr. Shute, no medical man, whatever his culture and capacity, need wish for. His children and his many friends mourn his loss; but the memory of his noble life can never be other than a pure and permanent pleasure.

